# Method for the Identification of Taxon-Specific *k*-mers from Chloroplast Genome: A Case Study on Tomato Plant (*Solanum lycopersicum*)

**DOI:** 10.3389/fpls.2018.00006

**Published:** 2018-01-17

**Authors:** Kairi Raime, Maido Remm

**Affiliations:** Department of Bioinformatics, Institute of Molecular and Cell Biology, University of Tartu, Tartu, Estonia

**Keywords:** *k*-mer-based method, taxon-specific *k*-mers, plant taxon identification, raw sequencing reads, *Solanum lycopersicum*

## Abstract

Polymerase chain reaction and different barcoding methods commonly used for plant identification from metagenomics samples are based on the amplification of a limited number of pre-selected barcoding regions. These methods are often inapplicable due to DNA degradation, low amplification success or low species discriminative power of selected genomic regions. Here we introduce a method for the rapid identification of plant taxon-specific *k*-mers, that is applicable for the fast detection of plant taxa directly from raw sequencing reads without aligning, mapping or assembling the reads. We identified more than 800 *Solanum lycopersicum* specific *k*-mers (32 nucleotides in length) from 42 different chloroplast genome regions using the developed method. We demonstrated that identified *k*-mers are also detectable in whole genome sequencing raw reads from *S. lycopersicum*. Also, we demonstrated the usability of taxon-specific *k*-mers in artificial mixtures of sequences from closely related species. Developed method offers a novel strategy for fast identification of taxon-specific genome regions and offers new perspectives for detection of plant taxa directly from sequencing raw reads.

## Introduction

The molecular identification of plant species is one possible approach when processed material has to be analyzed and the visual inspection of morphology is not possible. Using DNA-based tests for authentication plays an important role in the detection of mislabelled species and inadvertent or intentional species substitutions in food products (Galimberti et al., 2013; Ferri et al., 2015), detecting species and their origin in forensic samples in investigating of crimes (e.g., illegal trade of flora and fauna) (Iyengar, 2014), the identification of unlabelled herbal ingredients in herbal drugs (Newmaster et al., 2013) and the analysis of the composition of decayed plant material from soil, e.g., root or leaf litter samples (Wallinger et al., 2012).

DNA barcoding is a widely used method for the identification of plant, animal or fungi taxa by sequencing a standardized short DNA fragment. Barcoding methods identify taxa very efficiently within metagenomics samples of different origin (Coghlan et al., 2012; Newmaster et al., 2013; Tillmar et al., 2013; Zhou et al., 2013), but the success of the identification or detection of species depends on the selected loci. DNA barcodes usually constitute only a very small part of the genome (usually less than 1000 bp), and due to different limiting factors (e.g., low PCR efficiency, gene deletions, and insufficient variation of selected barcode regions), no single-locus barcode has been identified as a universal DNA barcode region for identification of all plants. Using multi-locus combinations of two or three loci has been suggested (Newmaster et al., 2006; Kress and Erickson, 2007; Cbol Plant Working, 2009; Mishra et al., 2016). Using only a few barcoding regions may still not be enough for differentiate phylogenetically close plant species (Clement and Donoghue, 2012; Zhang et al., 2012), but designing primers for the amplification of hundreds or thousands of different barcode regions is very time-consuming and costly.

DNA metabarcoding method bases on next-generation sequencing of pre-amplified DNA barcodes for identification of multiple taxa simultaneously from a single metagenomics sample and has also been applied for the identification of plant and animal species from the various metagenomics samples (Staats et al., 2016). However, the barcoding of samples containing multiple taxa can be complicated in samples with degraded DNA. Varied polymerase chain reaction (PCR) success for selected genes, gene copy numbers and PCR bias caused by primer-template mismatches across species may cause species to be missed and reduce the quantitative potential of the method (Elbrecht and Leese, 2015; Piñol et al., 2015).

DNA mini-barcodes with significantly reduced length barcode sequences (100–250 bp) have been introduced to improve PCR amplification success of samples with degraded DNA (Dong et al., 2014; Shokralla et al., 2015). Mini-barcodes from the chloroplast genome display very low inter-populational variations and are found to be almost free of intra-populational variations and, therefore, sequence divergence is predominantly between species. Taxa mini-barcodes with high resolution are not easily found (Dong et al., 2014). All PCR-based detection methods (including all barcoding methods) are able to detect only DNA from species to which PCR primers bind efficiently. Therefore, discriminating power of different barcoding methods is directly dependent on the selected barcode markers and reference database composition (Ficetola et al., 2010; Hollingsworth et al., 2011; Dong et al., 2014).

More innovative approaches for the identification of species from metagenomics samples include the deep sequencing of total genomic DNA from samples with various taxon composition. These potentially avoid some of the problems associated with targeted PCR-based methods. Therefore, these has also been suggested as a valuable tool for species identification and quantification in food testing (Ripp et al., 2014).

However, the taxonomic classification of metagenome sequencing reads can be challenging, especially when analyzing short reads derived from next generation sequencing. One approach for the identification of taxa is comparing the pre-assembled reads to the reference genomes. However, metagenomics assemblies and the quantification of taxa from contigs is computationally challenging and assembly free methods that are based on sequence alignment are too slow to cope with the increasing amount of available genomic data (Menzel et al., 2016).

Thus, the algorithms have been developed that are not using traditional alignment methods for the taxonomic classification of individual sequencing reads, but are based on the hash-based index structures created from a set of reference sequences. For the taxonomic assignment of the reads, all the *k*-mers (short exact-matching substrings of a fixed-length *k*) contained in the reference genomes are stored in an index for fast lookup and the *k*-mers in each sequencing read are searched in this index. The read is assigned to a taxon based on the matching genomes (Menzel et al., 2016). Recent programs following this approach are Kraken (Wood and Salzberg, 2014), Clark (Ounit et al., 2015), GenomeTester4 (Kaplinski et al., 2015), and Centrifuge (Kim et al., 2016). These programs do not require a genome assembly or mapping of the data to a reference genome. The analysis can be performed directly on sequencing reads and therefore has the potential to be less error-prone and faster than traditional methods (Patro et al., 2014; Wood and Salzberg, 2014; Kaplinski et al., 2015; Ounit et al., 2015). There are many examples for applications of *k*-mer-based methods in the detection of bacterial taxa in metagenomics samples (Wood and Salzberg, 2014; Ounit et al., 2015; Kim et al., 2016; Roosaare et al., 2017), but most of these are not developed or tested for the identification or detection of plant taxa in metagenomics samples. As a result of analysis of the sequencing reads of fruit shake containing more than a dozen plant species using Centrifuge (Kim et al., 2016) about half of the plant species were identified. Many plant species remained unidentified, probably because their genome sequences were substantially different compared to those in the database used for analysis or because the abundance of those plants was very low in the sample. There were also some problems with discriminating close species (e.g., apple and pear) (Kim et al., 2016).

More than 100 nuclear plant genomes have been sequenced and many more are expected in the years to come (Weigel and Mott, 2009; Michael and Jackson, 2013; Nystedt et al., 2013). However, unlike bacteria, the number of sequenced nuclear plant genomes is currently not sufficient for finding taxon-specific *k*-mers. Mitochondrial genomes from plants are a poor choice for finding taxon-specific DNA regions, because plant mitochondrial DNA evolves slowly in sequence (Palmer and Herbon, 1988; Drouin et al., 2008) and because of intra-individual variability (caused by heteroplasmy) (Kmiec et al., 2006).

The low variation in the plastid genes analyzed for DNA barcoding and the availability of more than 2500 sequenced chloroplast genomes from a variety of land plants has led to the idea of using entire plastid genome sequences to improve the resolution in resolving evolutionary relationships at lower taxonomic levels with limited sequence variation (Parks et al., 2009; Whittall et al., 2010; Nock et al., 2011; Zhang et al., 2011; Kane et al., 2012; Yang et al., 2013; Ruhsam et al., 2015). The size of most chloroplast genomes in higher plants ranges between 115 and 165 kb and show a high similarity in their structure and gene organization (Jansen et al., 2005). The chloroplast genome contains a large single-copy (LSC) and a small single-copy (SSC) region, which are separated by two copies of an inverted repeat (IR) (Sugiura et al., 1998). It is also known that IR-s are highly conserved among plants. The mutation rate and sequence variability in the single-copy regions of the plastome is higher than in the IR-s (Wolfe et al., 1987; Maier et al., 1995; Kahlau et al., 2006; Dong et al., 2012).

The advantage of using taxon-specific DNA *k*-mers found in the chloroplast genome for the identification of plant taxa from metagenomics samples is that the chloroplast genome is endemic to plants and may help to bypass DNA contamination from organisms without chloroplasts (e.g., animals and fungi) (Dong et al., 2014). Chloroplast genome-derived markers are reliable for the identification of plant species, as the chloroplast DNA is high copy and has small and generally stable, mechanical breakdown resistant, circular form compared to nuclear DNA (Kim et al., 2015).

In this work, we introduce a fast pipeline for the identification plant taxon-specific *k*-mers from the chloroplast genome, that can be used for the qualitative detection of plant taxa directly from raw sequencing data. We used the plant species *Solanum lycopersicum* (tomato plant) as an example for finding species-specific *k*-mers. We also analyzed the presence and number of taxon-specific *k*-mers (identified from chloroplast genome) in whole genome sequencing raw reads of different *Solanaceae* species.

## Results

### Pipeline for Selecting Taxon-Specific *k*-mers

**FIGURE 1:**
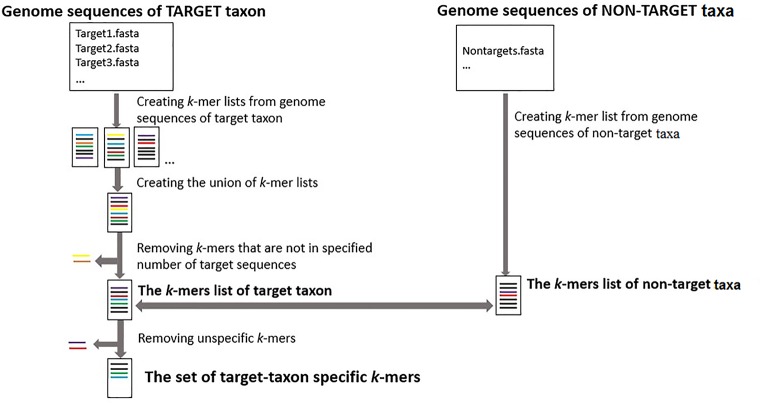
The workflow for identifying taxon-specific *k*-mers. The process of finding a set of taxon-specific *k*-mers starts with creating *k*-mer lists for each sequence of target taxon and all sequences of non-target taxa (from FASTA or FASTQ files). Next the set of *k*-mers that are not present in a specified number of target sequences or are also present in any non-target sequences are removed to identify the set of taxon-specific *k*-mers.

The first step for selecting the taxon-specific *k*-mers is creating the *k*-mer lists of every target sequence and *k*-mer list of all non-targets (**Figure 1**). Target sequences and non-target sequences used as inputs can be assembled chloroplast genome sequences or any other genome regions for target and non-target taxa.

The choice of the most appropriate *k* value (the length of oligomers) depends on the specific task. Reducing the length of *k*-mers when selecting taxon-specific *k*-mers would increase the number of *k*-mers that are present in the different target-taxon sequences, but with the cost of increasing the probability of finding those *k*-mers in sequences from non-target taxa (Ounit et al., 2015).

The next step is finding a union of unique target *k*-mers that are present in the specified number of target sequences. The final step is comparing the list of target *k*-mers to the list of non-targets *k*-mers to identify the final list of target taxon specific *k*-mers that are present in the specified numbers of target sequences but are not present in any non-target sequences. GenomeTester4 programs (Kaplinski et al., 2015) have been used for creating and comparing the *k*-mer lists.

### *Solanum lycopersicum* Specific *k*-mers

To test whether species-specific *k*-mers can be selected for real species, we tested our pipeline for the detection of *S. lycopersicum* specific *k*-mers using available and assembled chloroplast genome sequences.

Although the cultivated tomato plant, *S. lycopersicum*, has been a subject of extensive breeding programs, the nucleotide sequences of plastid genomes in different tomato varieties show very low sequence variation (Kahlau et al., 2006). The overall chloroplast genomic structures and sequences from different species in the *Solanaceae* family have been found to be quite similar, though an analysis of complete alignment of the chloroplast genome sequences identified a number of genetic variations in the intergenic spacer regions and protein-coding genes between different *Solanaceae* species (Chung et al., 2006). Complete chloroplast genome sequences have been rarely used to discriminate *Solanum* species (Gargano et al., 2012; Cho and Park, 2016).

**FIGURE 2:**
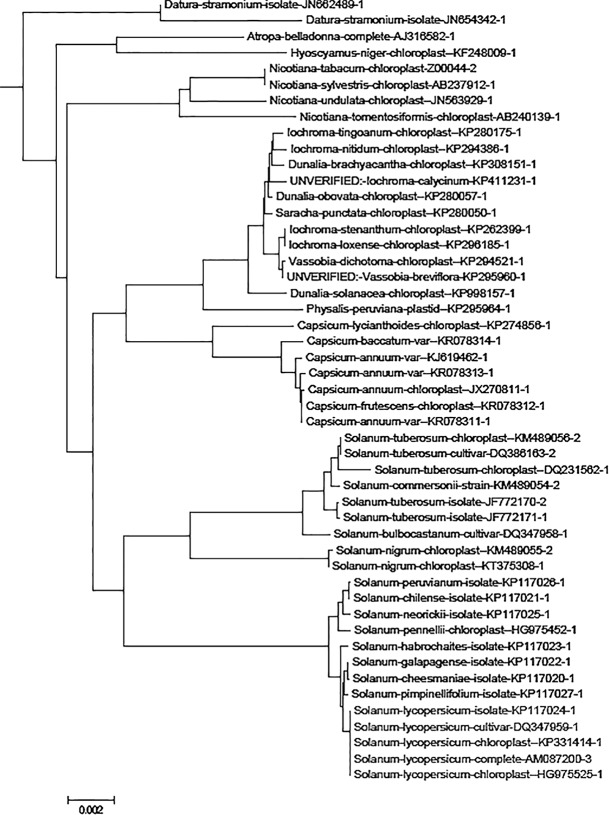
The *Solanaceae* family subtree in the tree containing 1,719 plant chloroplast genome sequences. The genome name contains the NCBI GenBank name and the accession number. The sequences *Solanum lycopersicum* species sequences are highlighted in blue and other sequences (non-target species) are indicated in black. The neighbor-joining tree was constructed using MEGA version 6 (Tamura et al., 2013) and is based on distance matrix created with andi (Haubold et al., 2015).

To get an overview of the available data for chloroplast genome sequences, we constructed a tree containing all 1,719 chloroplast genome sequences downloaded from the GenBank database. According to the tree, all five *S. lycopersicum* plastid genome sequences are in the same branch of the tree, and the *S. lycopersicum* sequences are the most closely related to *S. pimpinellifolium* (wild species of tomato plant) sequences (**Figure 2**).

**FIGURE 3:**
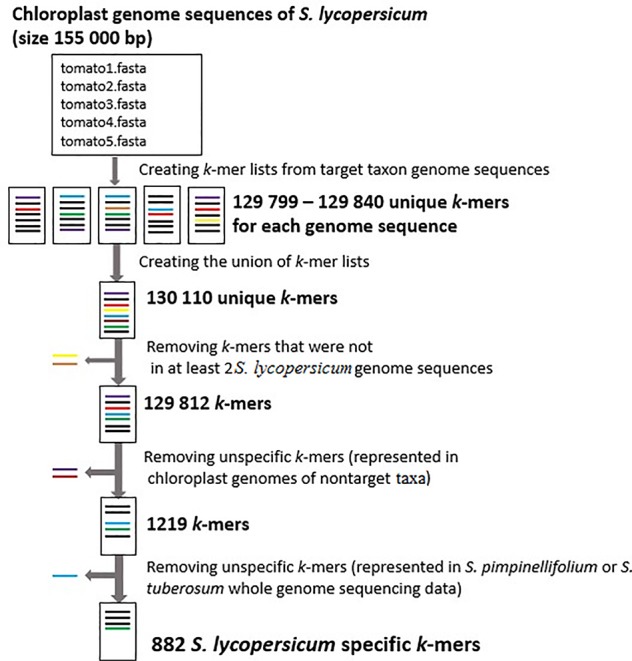
The number of *S. lycopersicum k*-mers in every step of the pipeline. Each FASTA file contains assembled chloroplast genome sequences from different *S. lycopersicum* samples. There were 129,799 – 129,840 unique *k*-mers (32 nucleotides in length) in each chloroplast genome sequence, 130,110 unique *k*-mers in the union of *k*-mers from all the sequences and 129,812 *k*-mers that were present in at least 2 *S. lycopersicum* chloroplast genome sequences. After removing the *k*-mers that were not present in at least two target sequences and unspecific *k*-mers that were present in assembled chloroplast genome sequences of non-target taxa or whole genome sequencing raw reads from *Solanum pimpinellifolium* and *Solanum tuberosum*, we detected 882 *S. lycopersicum* specific *k*-mers.

**FIGURE 4:**
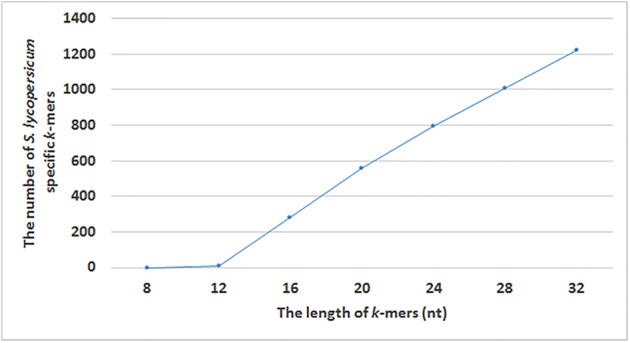
The number of identified *S. lycopersicum* specific *k*-mers with different lengths (8, 12, 16, 20, 24, 28, and 32 nucleotides).

Using our pipeline for the identification of *S. lycopersicum* specific *k*-mers from assembled chloroplast sequences in five *S. lycopersicum* as the target taxon sequences and 1,714 other plant species as the non-target taxa sequences, we detected 882 *S. lycopersicum* specific *k*-mers (32 nucleotides in length). The tomato plant chloroplast genome is approximately 155,000 bp and there were 129,812 *k*-mers that were present in at least two chloroplast genome sequences from *S. lycopersicum*. After removing the *k*-mers that were not present in at least 2 *S. lycopersicum* sequences (individual-specific *k*-mers) and the unspecific *k*-mers that were present in the assembled chloroplast genome sequences of non-target species or whole genome sequencing raw reads for *S. pimpinellifolium* and *S. tuberosum*, we detected 882 *S. lycopersicum* specific *k*-mers (**Figure 3**). We used *k*-mer length 32 nt, which gave us the maximum number of *S. lycopersicum* specific *k*-mers (**Figure 4**) for the identification of *S. lycopersicum* in sequencing raw reads.

### The Number of *Solanum lycopersicum* Specific *k*-mers Detected from Genomic Reads

To test whether *S. lycopersicum* specific *k*-mers (detected from plastid genome sequences) are detectable also in whole genome sequencing raw data, we analyzed the number of *S. lycopersicum* specific *k*-mers detected in whole genome sequencing raw reads from different *Solanaceae* species (*S. lycopersicum*, *S. pimpinellifolium, S. tuberosum*, *S. melongena*, and *Capsicum annuum*). The reads were downloaded from NCBI SRA database (details in the see section “Materials and Methods”). To also consider the influence of the number of sequencing reads on *k*-mers detection, we simulated new FASTQ files with different numbers of sequencing reads (10^3^–10^8^) from original FASTQ files for all the samples.

**FIGURE 5:**
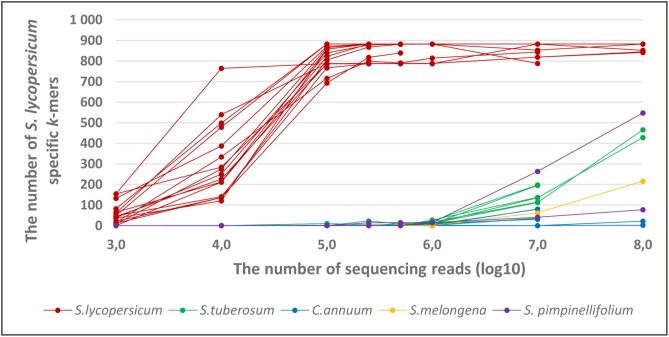
The number of detected *S. lycopersicum* specific *k*-mers in the whole genome sequencing raw data from *S. lycopersicum, S. pimpinellifolium*,*S. tuberosum, Solanum melongena*, and *Capsicum annuum* with variable number of sequencing reads (10^2^–10^8^). The set of taxon-specific *k*-mers contains 882 *k*-mers that were present in at least 2 *S. lycopersicum* chloroplast sequence. The samples from the *S. lycopersicum* target species are marked with a red color, and the non-target species are in different colors.

The results showed that *S. lycopersicum* specific *k*-mers, that originated from the chloroplast genome, are also detectable in whole genome sequencing raw reads from *S. lycopersicum.* The number of detected *S. lycopersicum* specific *k*-mers from *S. lycopersicum* sequencing data increased with the number of sequencing reads. Almost all 882 *k*-mers from the pre-selected set of taxon-specific *k*-mers were detected, when the number of sequencing reads was at least 10^5^ (**Figure 5**). Assuming that the sequencing coverage of different genomic regions is almost equal, 10^5^ sequencing reads provides coverage for *S. lycopersicum* nuclear genome of approximately 0,01×. The chloroplast genome coverage is difficult to estimate because of the variable copy number of plastome in individual plant cells.

In addition to the *S. lycopersicum* specific *k*-mer list containing *k*-mers that were present in at least 2 *S. lycopersicum* chloroplast genome sequences, we performed similar experiments with the *k*-mer lists that contained *S. lycopersicum* specific *k*-mers that were present in at least 1 or at least 5 (all) *S. lycopersicum* chloroplast sequences to analyze the influence of universality cut-off value on *k*-mers detectability. The results for the different sets were similar (Supplementary Figure 1).

To analyze the specificity of the *S. lycopersicum* specific *k*-mers, we analyzed the number of *S. lycopersicum* specific *k*-mers in the whole genome sequencing raw reads from phylogenetically close non-target taxa (*S. pimpinellifolium*, *S. tuberosum*, *S. melongena*, and *C. annuum*). The results showed that the number of detected *S. lycopersicum* specific *k*-mers in whole genome sequencing raw data from phylogenetically close species started to increase as the read number exceeded 10^6^, it reached to 548 in the whole sequencing raw data from *S. pimpinellifolium* when the sequencing read number was 10^8^ (**Figure 5**). Therefore, for a metagenomics sample with an unknown proportion of *S. lycopersicum* and other taxa (e.g., *S. pimpinellifolium* or *S. tuberosum*), detecting less than 550 *S. lycopersicum* specific *k*-mers will generate complications for determine if these *k*-mers originate from *S. lycopersicum* or from other *Solanaceae* species.

We also used our method to identify species-specific *k*-mers for *Oryza sativa* and *Zea mays* and analyzed the number of *O. sativa* and *Z. mays* specific *k*-mers detected in whole genome sequencing raw reads from different samples of target taxa and also phylogenetically close non-target taxa (Supplementary Figure 2).

### Using the Frequency of *Solanum lycopersicum* Specific *k*-mers Detected in Genomic Reads to Increase Specificity

In the detection of taxon-specific *k*-mers from whole genome sequencing reads, in addition to the detection of the presence or absence of these *k*-mers (binary YES/NO detection, *k*-mer was detected if it was represented in the sample with frequency at least 1), it would be helpful, if we could also consider the frequency of every *k*-mer in the sequencing reads. Although, the copy numbers of chloroplast genome may vary in different samples, we assumed that all the *k*-mers from the *S. lycopersicum* chloroplast genomes are represented in raw sequencing reads from one sample with similar frequency, which reflects the copy numbers of chloroplast genomes in the sample. This could help to distinguish the *k*-mers truly from *S. lycopersicum* (should be with similar frequency) and *k*-mers from close non-target species (caused by sequencing errors etc.).

**FIGURE 6:**
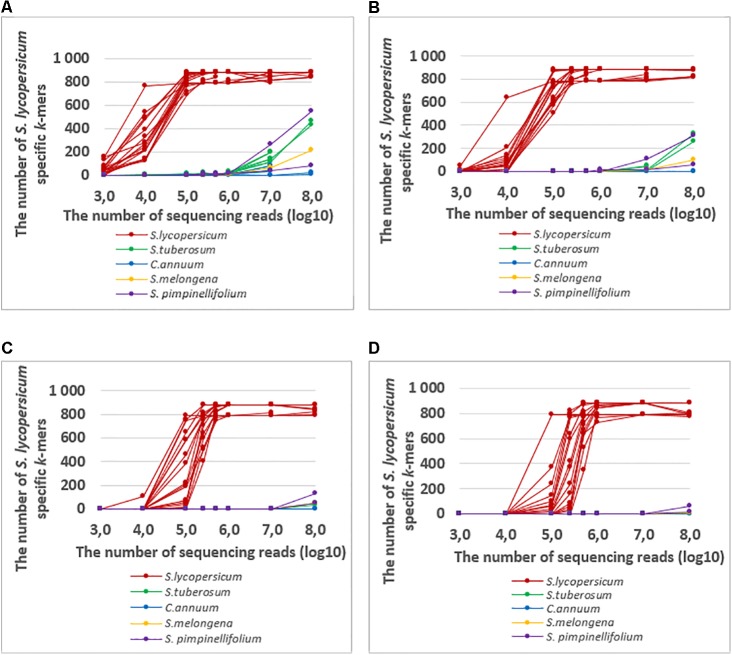
The numbers of detected *S. lycopersicum* specific k-mers with frequencies of at least **(A)** 1, **(B)** 2, **(C)** 5, and **(D)** 10 in the whole genome sequencing raw data from *S. lycopersicum, S. pimpinellifolium, S. tuberosum, S. melongena*, and *C. annuum* with variable numbers of sequencing reads (10^3^–10^8^). The detected *S. lycopersicum* specific *k*-mers were present in at least 2 *S. lycopersicum* chloroplast sequences.

Assuming that *k*-mers with very low frequency can be the result of sequencing errors, we tried to increase the *k*-mer frequency cut-off value (from 1 to 10) to increase the specificity. The increased cut-off value decreased the number of detected *S. lycopersicum* specific *k*-mers in *S. tuberosum* and *S. pimpinellifolium*, even when the read number was more than 10^7^ (specificity increased). However, at in the same time we were not able to detect *S. lycopersicum* specific *k*-mers in some of the *S. lycopersicum* samples when the sequencing read number was 2.5^∗^10^5^ or less (sensitivity of *S. lycopersicum* detection decreased) (**Figure 6**). Therefore, it is possible to increase the specificity but with the price of sensitivity. Thus the appropriate choice of the frequency cut-off value depends on the specific case.

### The Location of the *Solanum lycopersicum* Specific *k*-mers in the Chloroplast Genome

**FIGURE 7:**
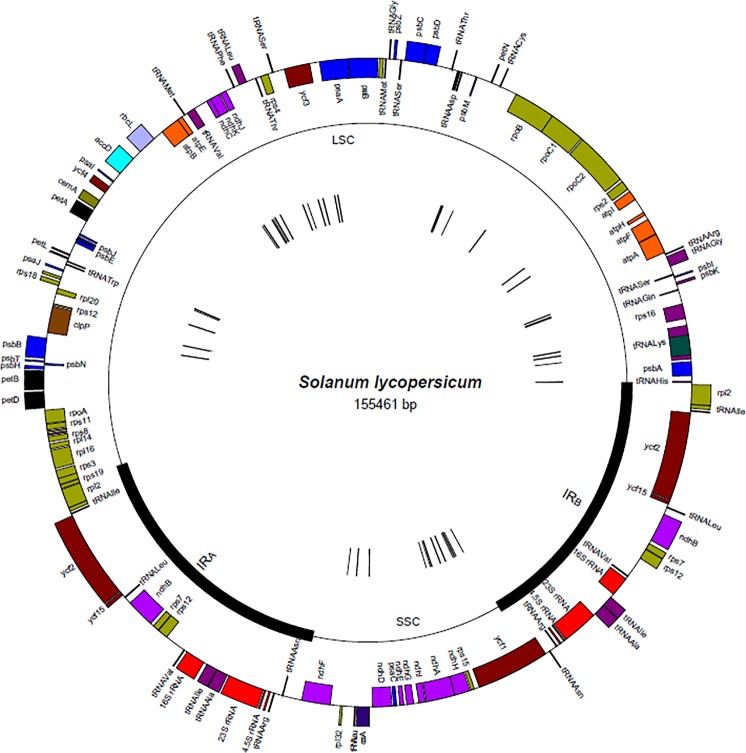
The location of the *S. lycopersicum* specific k-mer clusters in the chloroplast genome (in sequence NC_007898.3). The outer circle illustrates the location of the chloroplast genes and the middle circle indicates the location of the inverted repeats (IR_A_ and IR_B_) and single copy regions (SSC and LSC). The location of the 42 different clusters of *S. lycopersicum* specific k-mers are shown as small black strokes in the inner circle in the figure.

The locations of all 882 *S. lycopersicum* specific *k*-mers identified in this work using our pipeline are described (in **Figure 7**). The *S. lycopersicum* specific *k*-mers were located along the entire chloroplast genome as 42 clustered taxon-specific regions along the single copy regions in the *S. lycopersicum* chloroplast genome (most of them were located in intergenic spacer regions or introns). The *k*-mers that were located adjacent to each other and differ by only one nucleotide belonged in the same cluster. The sequences in the *k*-mers clusters contain at least 1 mismatch per 32 bp of *S. lycopersicum* sequence compared to all non-target sequences.

The lengths of the 42 detected taxon-specific regions in the *S. lycopersicum* chloroplast genome varied from 33 to 62 bp (average 53.3 bp, median 57.5 bp), meaning that there was 2–31 *k*-mers in one cluster. The sequence lengths bp) between two adjacent cluster regions (region between the end of the last *k*-mer from one cluster and the start of the first *k*-mer sequence from the next cluster) varied from 30 to 39,507 bp.

## Discussion

Current developments in the identification plant taxa in degraded metagenomics samples are moving toward using a combination of many different DNA barcodes with reduced lengths (e.g., mini-barcodes) (Dong et al., 2014; Shokralla et al., 2015; Wang et al., 2016) and deep sequencing of total genomic DNA from samples with various taxon composition, followed by the identification of the taxonomical origin of the sequencing reads (Ripp et al., 2014). *k*-mer based methods, that do not require prior primer design and the pre-amplification of specific regions, genome assembly and mapping of sequencing reads to a reference genome, have already been applied for the identification of microbial species or strains in metagenomics samples (Wood and Salzberg, 2014; Ounit et al., 2015; Kim et al., 2016; Roosaare et al., 2017).

Here we introduce a *k*-mers-based approach to detect plant taxa from raw sequencing reads. We showed that it is possible to identify a set of plant taxon-specific *k*-mers from the chloroplast genome, that are also detectable from whole genome sequencing raw reads of target taxon. Different pieces of available softwares enabled fast identification and counting of taxon-specific *k*-mers from genome sequences from different taxa (e.g., Kaplinski et al., 2015).

To find a correct set of taxon-specific *k*-mers, it is recommended to consider also taxonomic ambiguity and use sequences from a reliable database, where the reference specimen has been correctly identified, because several gaps and false sequences could influence the results. The availability of many reliable genome sequences for a target taxon as well as many sequences for phylogenetically close non-target taxa are good presumptions for finding target taxon-specific *k*-mers. The number of available plant whole genome sequences is still not sufficient for finding taxon-specific *k*-mers, but there are many advantages to use chloroplast genome sequences for finding taxon-specific *k*-mers for detection of pre-selected plant taxon from metagenomics sample (e.g., high copy number, interspecific variability, low intraspecific variability, consistently increasing number of available sequences). To get overview of the available data of target and non-target sequences we constructed a tree containing more than 1,700 chloroplast genome sequences.

Although the variability of chloroplast genome sequences between very close plant species can be quite low and finding barcoding regions for amplification has been restricted (Fazekas et al., 2008; Hollingsworth et al., 2011), we identified a set of *S. lycopersicum* specific *k*-mers from the chloroplast genome. The set of taxon-specific *k*-mers can be identified from the chloroplast genome using available software in only a few minutes, and the set of taxon-specific *k*-mers can be easily updated if additional genomic sequences are available in biological databases for either target or non-target species.

Using assembled chloroplast sequences from the GenBank database, we detected 882 *S. lycopersicum* specific *k*-mers that were 32 nucleotides in length (excluding individual-specific *k*-mers). Our results showed that the identified *S. lycopersicum* specific *k*-mers were located in 42 different regions along the *S. lycopersicum* chloroplast genome single copy regions, which is in accordance with the previous findings that the mutation rate in the plastome single copy regions is much higher than in the IR regions (Wolfe et al., 1987; Maier et al., 1995; Kahlau et al., 2006). None of the *k*-mers were in *S. lycopersicum* chloroplast genome IR regions, which is probably due to the low mutation rate and therefore very few differences between the sequences in the IR regions of very close plant species. The detected *k*-mers were most frequently in intergenic spacer regions or introns. Previous studies have identified the following thirteen most variable plastid marker regions in *Solanum* species: atpB-rbcL, clpP-psbB, ndhF, ndhF-rpl32, petL-psaJ (including petL-petG-trnW and trnP-psaJ), petN-psbM, rpl32-trnL, rpoC1-rpoB, trnA-trnI, trnK-rps16, and ycf1 (parts 1–3) (Särkinen and George, 2013). Most of these are also regions that contain *S. lycopersicum* specific *k*-mers identified in our study.

The detection of pre-selected *S. lycopersicum* specific *k*-mers in the whole genome sequencing raw reads from *S. lycopersicum* showed that *k*-mers from chloroplast genomes are also detectable from whole genome sequencing raw reads, and the number of detected *S. lycopersicum* specific *k*-mers increases with the increase in the number of sequencing reads. Almost all 882 k-mers were detected when read number was at least 100,000.

Most of the *S. lycopersicum* specific *k*-mers differed from *S. pimpinellifolium* or *S. tuberosum* chloroplast genome sequences by only one nucleotide. Therefore, probably due to sequencing errors (Nakamura et al., 2011; Liu et al., 2012), the number of detected *S. lycopersicum* specific *k*-mers also increased in the samples from other *Solanaceae* species when the read number was greater than 1,000,000. This could be a particular challenge when detecting tomato plant metagenomics samples containing large amounts of sequencing reads from phylogenetically close non-target taxa (like currant tomato or potato) and small amount of reads from *S. lycopersicum*. However, it would be possible to detect *S. lycopersicum* from raw sequencing reads using taxon-specific *k*-mers from the chloroplast genome, even from metagenomics samples containing tomato, currant tomato, potato, eggplant, and pepper, if the read number from *S. lycopersicum* is at least 100,000 and approximately 600 *k*-mers (from 882 *S. lycopersicum* specific *k*-mers) are detected.

It is difficult to take into account the frequency of detected *k*-mers to increase the specificity or to estimate the amount of *S. lycopersicum* in a sample, as the frequency of *k*-mers from the chloroplast genome is influenced on many factors (organization of chloroplast genome, the copy number of chloroplasts and chloroplast genomes in different cells, in different parts of plants, depending on plant species, growth conditions, etc.) (Oldenburg and Bendich, 2004; Liere and Börner, 2013).

To find ways to increase the detection specificity while also considering the frequency of detected *k*-mers, we analyzed the frequency distribution of *S. lycopersicum* specific *k*-mers in different *S. lycopersicum* and other *Solanaceae* species samples. Although the average frequency varied between different samples, the overall frequency of detected *S. lycopersicum k*-mers in *S. lycopersicum* samples was substantially higher than in other (non-target) *Solanaceae* samples. Using increased *k*-mer frequency cut-off values increased the specificity (the number of detected *S. lycopersicum* specific *k*-mers in *S. tuberosum* and *S. pimpinellifolium* was much lower, even when the read number was more than 10^7^), though the sensitivity of *S. lycopersicum* detection was decreased (we were not able to detect *S. lycopersicum* specific *k*-mers in some of the *S. lycopersicum* samples if the sequencing read number was 2.5^∗^10^5^ or less). Therefore, the appropriate choice for the frequency cut-off value depends on the specific case.

The increasing number of available plant whole genome sequences probably in the future gives opportunity to identify additional plant taxa specific *k*-mers from nuclear genomes which would be useful, when discriminating very close species, subspecies or cultivars with very little or lack of chloroplast genome sequence variability.

Using hundreds of taxon-specific short *k*-mers from all over the genome for the identification or qualitative detection of plant taxa would give improved resolution at the species level as well as aid in analyzing complex and degraded samples that sequence barcoding or other traditional methods fail to resolve. However, to apply the identified *S. lycopersicum* specific *k*-mers to detect *S. lycopersicum* (tomato plant) from real metagenomics samples (food, medicines, environmental samples, etc.), additional testing with real metagenomics samples is required.

## Conclusion

Accurate and fast methods for the identification of plant(s) from degraded metagenomics samples play an important role in identifying the composition of complex mixtures of processed biological materials, including food, herbal products, gut contents, environmental samples, etc., PCR and different barcoding methods commonly used for plant identification are based on the amplification of a limited number of pre-selected barcoding regions. These methods are often inapplicable due to the degree of DNA degradation, low PCR amplification success or low species discriminative power of the selected barcoding regions.

Our method enables rapid identification of plant taxon-specific *k*-mers from the chloroplast genome. By applying our method to select a set of *S. lycopersicum* (tomato plant) specific *k*-mers, we identified 882 *S. lycopersicum* specific *k*-mers that were present in at least two chloroplast genome sequences from *S. lycopersicum* and none of the 1,714 chloroplast sequences from non-target species. *In silico* experiments using raw sequencing data from the *S. lycopersicum, S. tuberosum* (potato), *S. pimpinellifolium* (currant tomato), *S. melongena* (eggplant), and *C. annuum* (pepper) whole genomes showed that *S. lycopersicum* specific *k*-mers found in the chloroplast genome can also be detected from *S. lycopersicum* whole genome sequencing raw reads as well as in other *Solanaceae* species. If sequencing data from metagenomic samples contain at least 10^5^ reads from the *S. lycopersicum* genome, it is possible to discriminate *k*-mers from *S. lycopersicum* and *k*-mers from other *Solanaceae* species.

We are providing a valuable method here for the identification of plant taxon-specific *k*-mers that could be used in the future for developing diagnostic tests for the fast detection of different plant taxa in raw sequencing reads from metagenomics samples.

## Materials and Methods

### Identification of Taxon-Specific *k*-mers

By combining different programs (GListMaker, GListCompare, and GListQuery) from the GenomeTester4 software package (Kaplinski et al., 2015) and in-house scripts, we constructed *k*-mer analysis pipeline for finding a set of taxon-specific *k*-mers. GListMaker converts assembled chloroplast sequences or raw sequencing reads (FASTA or FASTQ) from the sample to *k*-mer lists and GListCompare compares different lists of *k*-mers to find intersections, differences or unions between the lists for detecting a set of taxon-specific *k*-mers.

### Identification of *Solanum lycopersicum* (Tomato Plant) Specific *k*-mers

To get an overview of the available data from assembled chloroplast genome sequences, we constructed a neighbor-joining tree consisting of 1,719 chloroplast genome sequences from plants and some green algae including 17 sequences from *Solanum* species, and five sequences from *S. lycopersicum*, which were downloaded from the GenBank database (Clark et al., 2016) (the accession numbers of complete chloroplast genome sequences we used to find *k*-mers are in Supplementary Data 1). The tree was constructed using the computer program MEGA version 6 (Tamura et al., 2013) and was based on a distance matrix constructed using the computer program andi (Haubold et al., 2015) to get an overview of the available data. The phylogenetic tree of *Solanaceae* is a subtree from the constructed tree.

To identify *S. lycopersicum* specific k-mers, we used different GenomeTester4 programs and in-house scripts (pipeline is available in the public repository Github). We created a *k*-mer list for *S. lycopersicum* and a *k*-mer list for other (non-target) species. The final set of *S. lycopersicum* specific *k*-mers containing 882 *k*-mers was identified using two steps for removing non-specific *k*-mers from the *S. lycopersicum k*-mer list. The first step used assembled plastome sequences from non-target taxa to remove non-specific *k*-mers that were represented in the chloroplast genome of non-target taxa. The second step used whole genome sequencing raw data from the two phylogenetically close non-target species *S. pimpinellifolium* and *S. tuberosum* with a frequency at least 10 to remove additional non-specific *k*-mers that may be represented in the nuclear or mitochondrial genomes of non-target taxa *S. pimpinellifolium* and *S. tuberosum*. A cut-off value of 10 removed most of the non-specific *k*-mers present in the nuclear, mitochondrial or chloroplast genome regions of the non-target taxa, but did not include *k*-mers caused by sequencing errors in the sequencing reads. The raw sequencing reads from 1 *S. pimpinellifolium* and 3 *S. tuberosum* (ERR418080, SRR1608100, SRR2069941, and SRR1481624) were downloaded from the NCBI SRA database (Leinonen et al., 2011).

In our study, we made analyses with different *k*-mer lengths to select the *k* value that gives the maximum number *of S. lycopersicum* specific *k*-mers, that can next be used for the identification of *S. lycopersicum* in sequencing raw reads. We used *k* values 8, 12, 16, 20, 24, 28, and 32 for finding taxon-specific *k*-mers for *S. lycopersicum*. The appropriate length of the *k*-mer depends on the taxon or the taxonomic level we were trying to find specific *k*-mers for and on the variability of the used genomic sequences. Smaller *k*-mer length leads to an increased number of *k*-mers that are represented in all sequences from a target taxon, but to the lower specificity of those *k*-mers (Ounit et al., 2015). Working with *k*-mers up to *k* = 32 is more efficient on 64-bit computers than using longer *k*-mers. GenomeTester4 programs used in our pipeline are summarizing the frequencies of the *k*-mer and its reverse complement *k*-mer when creating *k*-mers’ lists, i.e., one *k*-mer in a stored list represent both a *k*-mer and its reverse complement (Kaplinski et al., 2015). Therefore, it is not important to distinguish *k*-mers with odd and even length or prefer only odd *k*-mers.

We created the following three lists of *S. lycopersicum* specific *k*-mers: (1) *k*-mers that were present in all five *S. lycopersicum* chloroplast sequences, (2) *k*-mers that were present at least in 2 *S. lycopersicum* chloroplast sequences, and (3) *k*-mers that were present at least 1 *S. lycopersicum* chloroplast sequence. We selected one of these lists of *k*-mers for the subsequent analysis.

### *Solanum lycopersicum* Specific *k*-mers in Whole Genome Sequencing Raw Reads from Different *Solanaceae* Species

We used gmer_counter (Pajuste et al., 2017) and different Python scripts to detect of *S. lycopersicum* specific *k*-mers in the raw sequencing reads from whole genome samples of the following different *Solanaceae* species: (1) 16 *S. lycopersicum* (tomato), (2) 10 *S. tuberosum* (potato), (3) 4 *C. annuum* (pepper), (4) 2 *S. pimpinellifolium* (currant tomato), and (5) 1 *S. melongena* (eggplant) (ERR539598, SRR1572263, ERR418062, ERR418047, DRR000741, DRR040154, DRR040149, DRR040095, ERR418079, ERR418069, SRR1572661, SRR1572551, SRR404081, DRR022703, DRR022708, ERR964441, SRR307673, SRR2069932, SRR2070065, SRR1501269, SRR2069942, SRR1608091, SRR1 607674, SRR1525230, SRR1594256, ERR023052, SRR2752033, SRR2752003, SRR653457, SRR653499, ERR418082, SRR074949, and DRR014074). All the datasets downloaded from NCBI SRA database (Leinonen et al., 2011) contained whole genome sequencing raw reads. The sequencing read lengths in these datasets were from about 80 to 500 bp, but predominantly 200 bp, DNA was extracted mostly from the plant leaves or from tuber (potato).

To analyze the relationship between the number of detected *S. lycopersicum* specific *k*-mers and the number of next-generation sequencing reads, we simulated new FASTQ files with different number of reads (10^3^, 10^4^, 10^5^, 2.5^∗^10^5^, 5^∗^10^5^, 10^6^, 10^7^, and 10^8^) from the raw sequencing data files for all the samples. The reads were evenly selected from an original FASTQ file [downloaded from NCBI SRA database (Leinonen et al., 2011), i.e., when creating the new FASTQ file with 100,000 reads, every 1000. read from the original FASTQ file with 100,000,000 reads goes to the new FASTQ filean in-house developed Python script was used for this]. Therefore, different new FASTQ files for the same sample with different numbers of sequencing reads may not contain exactly the same set of reads.

We used four different cut-off values (1, 2, 5, and 10) for the frequency of the detected *k*-mers to analyse the influence of increased cut-off values on the specificity and sensitivity of *S. lycopersicum* detection from raw sequencing reads using pre-selected *S. lycopersicum* specific *k*-mers.

We also analyzed the frequency of individual *k*-mers from the final set of *S. lycopersicum* specific *k*-mers in raw sequencing reads from 16 different *S. lycopersicum* and 17 other plant species whole genomes.

### The Location of *Solanum lycopersicum* Specific *k*-mers in the Chloroplast Genome

The locations of the taxon-specific *k*-mers in the chloroplast genome were found using the *S. lycopersicum* chloroplast sequence NC_007898.3 using Python scripts. A physical map of the tomato plant plastome was drawn using the GenomeVX software (Conant and Wolfe, 2008).

## Availability of Data and Material

The datasets analyzed in the current study are available in the NCBI SRA (Sequence Read Archive), https://www.ncbi.nlm.nih.gov/sra, and in GenBank, https://www.ncbi.nlm.nih.gov/Genbank. The full list of accession numbers for the used sequences are given in Supplementary Data 1. The sequences of the *S. lycopersicum* specific *k*-mers identified in the current study are available from the corresponding author upon reasonable request. The pipeline, in-house scripts, including used parameters, are available in the public repository Github: https://github.com/bioinfo-ut/PlantTaxSeeker.

## Author Contributions

KR constructed the *k*-mer detection pipelines, wrote in-house Python scripts, analyzed the sequence data and wrote the manuscript. KR and MR designed the experiments and interpreted the results of all analysis. MR edited the manuscript. All authors read and approved the final manuscript.

## Conflict of Interest Statement

The authors declare that the research was conducted in the absence of any commercial or financial relationships that could be construed as a potential conflict of interest.

## Abbreviations

bp: base pair
NCBI: National Center for Biotechnology Information
SRA: Sequence Read Archive
