# Supplementary material for: Method for the Identification of Taxon-Specific k-mers from Chloroplast Genome: A Case Study on Tomato Plant (Solanum lycopersicum)
Source: Front Plant Sci. 2018 Jan 17;9:6. doi: 10.3389/fpls.2018.00006 (PMC5776150; doi:10.3389/fpls.2018.00006)
Supplement: Supplementary file 2 [file Supplementary_Figure_1.docx]

**A**

**B**

Supplementary Figure 1. The number of detected *Solanum lycopersicum* specific *k*-mers in whole genome sequencing raw data from *Solanum lycopersicum, Solanum pimpinellifolium, Solanum tuberosum, Solanum melongena* and *Capsicum annuum* with variable number of sequencing reads (10^2^-10^8^). The sets of *S. lycopersicum* specific kmers contained **(A)** 1096 *k*-mers that were present in at least 1 *S. lycopersicum* chloroplast genome sequence and **(B)** 851 *k*-mers that were present in at least 5 *S. lycopersicum* chloroplast genome sequences. The samples of target species *S. lycopersicum* are marked with a red colour and the non-target species with other colours.
