# Supplementary material for: Method for the Identification of Taxon-Specific k-mers from Chloroplast Genome: A Case Study on Tomato Plant (Solanum lycopersicum)
Source: Front Plant Sci. 2018 Jan 17;9:6. doi: 10.3389/fpls.2018.00006 (PMC5776150; doi:10.3389/fpls.2018.00006)
Supplement: Supplementary file 3 [file Supplementary_Figure_2.DOCX]

**(A)**


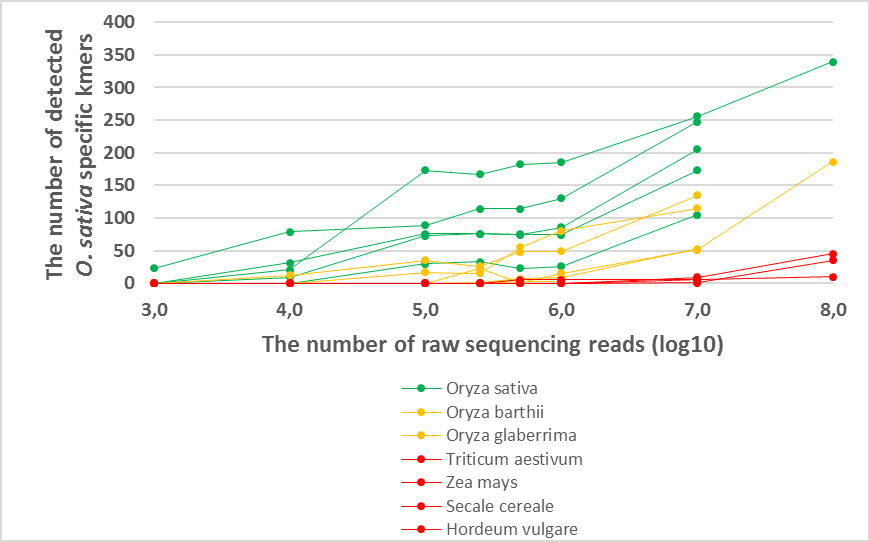


**(B)**


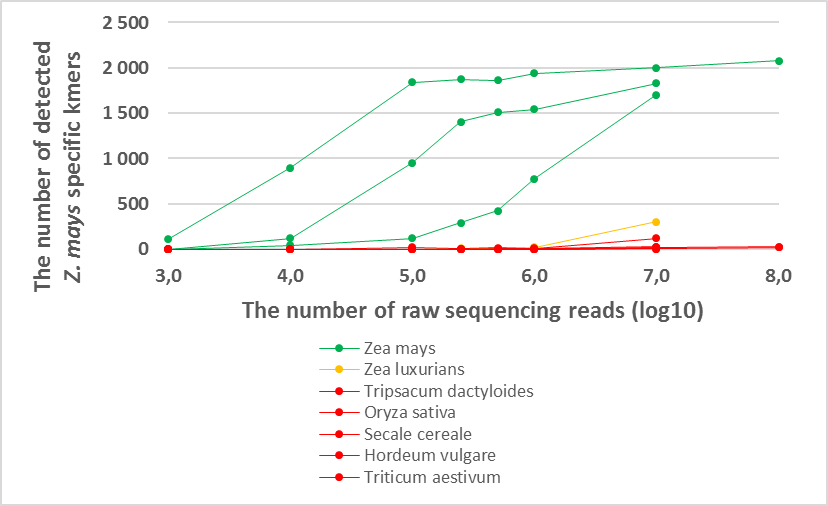


Supplementary Figure 2. The number of detected *Oryza sativa* (A) and *Zea mays* (B) specific *k*-mers in whole genome sequencing raw data from *O. Sativa* and *Z. mays* and some phylogenetically close nontarget species with variable number of sequencing reads (10^2^-10^8^). The set of *O. sativa* specific kmers contained 555 *k*-mers (A) and the set of *Z. mays* specific *k*-mers contained 2304 *k*-mers that were present in at least 2 target species chloroplast genome sequences. The samples of target species are marked with a red colour, nontarget species from the same genus with yellow and the other non-target species with red colour.

For the identification of *O. sativa* specific *k*-mers assembled chloroplast genome sequences of 17 *O. sativa* and non-target taxa (downloaded from Genbank database 06. Oct. 2017) were used. For the identification of *Zea mays* specific *k*-mers, assembled chloroplast genome sequences of 10 *Z. mays* and non-target taxa (downloaded from Genbank database 12. Oct. 2017) were used. Taxon-specific *k*-mers are represented in at least 2 target taxon sequences and none of the non-target sequences.

The accession numbers of FASTQ files downloaded from NCBI SRA database and used for analysis are following: SRR5951757, SRR5887649, SRR5886626, SRR5513409, SRR5877442 (*Oryza sativa*); SRR1206372, SRR1427734, DRR057972 (Oryza barthii); SRR1206511, SRR1206519 (Oryza glaberrima); SRR513014, SRR448925, SRR448388 (Zea mays), SRR088692 (Zea luxurians); ERR1706965 (Triticum aestivum); ERR505006 (Secale cereale); ERR246492 (Hordeum vulgare); SRR5121191, SRR4302006 (Tripsacum dactyloides).
